# Canadian Consensus Recommendations on the Management of KRAS G12C-Mutated NSCLC

**DOI:** 10.3390/curroncol30070476

**Published:** 2023-07-06

**Authors:** Parneet K. Cheema, Shantanu O. Banerji, Normand Blais, Quincy S.-C. Chu, Rosalyn A. Juergens, Natasha B. Leighl, Adrian Sacher, Brandon S. Sheffield, Stephanie Snow, Mark Vincent, Paul F. Wheatley-Price, Stephen Yip, Barbara L. Melosky

**Affiliations:** 1 Division of Medical Oncology, William Osler Health System, University of Toronto, Brampton, ON L6R 3J7, Canada; 2 Faculty of Medicine, University of Toronto, Toronto, ON M5S 1A8, Canada; 3 CancerCare Manitoba Research Institute, Department of Internal Medicine, Rady Faculty of Health Sciences, University of Manitoba, Winnipeg, MB R3E 0V9, Canada; sbanerji@cancercare.mb.ca; 4 Department of Medicine, Centre Hospitalier de l’Université de Montréal, University of Montreal, Montreal, QC H2X 3E4, Canada; normand.blais.med@ssss.gouv.qc.ca; 5 Division of Medical Oncology, Department of Oncology, Cross Cancer Institute, University of Alberta, Edmonton, AB T6G 1Z2, Canada; quincy.chu@albertahealthservices.ca; 6 Department of Medical Oncology, Juravinski Cancer Centre, McMaster University, Hamilton, ON L8V 5C2, Canada; juergensr@hhsc.ca; 7 Department of Medicine, Princess Margaret Cancer Centre, University Health Network, University of Toronto, Toronto, ON M5S 1A8, Canada; natasha.leighl@uhn.ca (N.B.L.); adrian.sacher@uhn.ca (A.S.); 8 Department of Laboratory Medicine, William Osler Health System, Brampton, ON L6R 3J7, Canada; 9 Division of Medical Oncology, Department of Medicine, QEII Health Sciences Centre, Dalhousie University, Halifax, NS B3H 2Y9, Canada; stephanie.snow@nshealth.ca; 10 Department of Medical Oncology, London Regional Cancer Program, London, ON N6A 5W9, Canada; mark.vincent@lhsc.on.ca; 11 Department of Medicine, The Ottawa Hospital Research Institute, The Ottawa Hospital, University of Ottawa, Ottawa, ON K1H 8L6, Canada; pwheatleyprice@toh.ca; 12 BC Cancer, Vancouver, University of British Columbia, Vancouver, BC V6T 1Z4, Canada; syip-02@bccancer.bc.ca; 13 Department of Medical Oncology, BC Cancer-Vancouver Centre, Vancouver, BC V5Z 4E6, Canada; bmelosky@bccancer.bc.ca

**Keywords:** KRAS G12C, NSCLC, targeted therapy, immune checkpoint inhibitors, mutation testing, resistance

## Abstract

Activating mutations in *Kirsten rat sarcoma viral oncogene homologue (KRAS)*, in particular, a point mutation leading to a glycine-to-cysteine substitution at codon 12 (G12C), are among the most frequent genomic alterations in non-small cell lung cancer (NSCLC). Several agents targeting KRAS G12C have recently entered clinical development. Sotorasib, a first-in-class specific small molecule that irreversibly inhibits KRAS G12C, has since obtained Health Canada approval. The emergence of novel KRAS-targeted therapies warrants the development of evidence-based consensus recommendations to help clinicians better understand and contextualize the available data. A Canadian expert panel was convened to define the key clinical questions, review recent evidence, and discuss and agree on recommendations for the treatment of advanced *KRAS G12C*-mutated NSCLC. The panel agreed that testing for KRAS G12C should be performed as part of a comprehensive panel that includes current standard-of-care biomarkers. Sotorasib, the only approved KRAS G12C inhibitor in Canada, is recommended for patients with advanced KRAS G12C-mutated NSCLC who progressed on guideline-recommended first-line standard of care for advanced NSCLC without driver alterations (immune-checkpoint inhibitor(s) [ICIs] +/− chemotherapy). Sotorasib could also be offered as second-line therapy to patients who progressed on ICI monotherapy that are not candidates for a platinum doublet and those that received first-line chemotherapy with a contraindication to ICIs. Preliminary data indicate the activity of KRAS G12C inhibitors in brain metastases; however, the evidence is insufficient to make specific recommendations. Regular liver function monitoring is recommended when patients are prescribed KRAS G12C inhibitors due to risk of hepatotoxicity.

## 1. Introduction

The discovery of several genomic alterations sensitive to targeted therapies has led to significant advances in the diagnosis and treatment of non-small-cell lung cancer (NSCLC). Yet, lung cancer remains one of the leading causes of cancer-related mortality [1], indicating the need for additional efforts to combat the disease. Activating mutations in *Kirsten rat sarcoma viral oncogene homologue (KRAS)*, in particular, a point mutation leading to a glycine-to-cysteine substitution at codon 12 (G12C), are among the most frequent genomic alterations in NSCLC [2].

*KRAS* mutations, including *KRAS G12C*, have traditionally been considered “undruggable” due to their intrinsic characteristics, lack of well-defined binding pockets, and complexity of their downstream signaling pathways. The new discovery of the allosteric site of KRAS G12C, however, has enabled the development of irreversible covalent inhibitors.

In September 2021, Health Canada approved the KRAS G12C inhibitor sotorasib for the treatment of adult patients with KRAS-G12C-mutated locally advanced or metastatic NSCLC who have received at least one prior systemic therapy [3]. Health Canada based the approval on results from the phase 2 CodeBreaK 100 trial [4]. Since then, data from the phase 3 clinical trial have been reported [5], and another KRAS G12C inhibitor, adagrasib, reached phase 3 clinical development (NCT04685135) [6]. Furthermore, several other KRAS inhibitors are in clinical development and will likely reach routine practice in the next few years.

As more therapeutic options are being investigated in patients with KRAS-mutant NSCLC, particularly those harbouring the *KRAS G12C* variant, it is critical to develop evidence-based consensus recommendations and generate novel therapeutic algorithms to improve patient selection and inform treatment decisions. Furthermore, due to the availability of other therapeutic options, including immune checkpoint inhibitors (ICIs) and cytotoxic chemotherapy, there is an added need for expert guidance on therapeutic approaches and sequencing of available therapies to optimize patients’ short- and long-term outcomes. Lastly, the therapeutic management of patients with advanced NSCLC and actionable genomic alterations likely differs across Canada due to variations in access to molecular testing and drug funding—further highlighting the need for such recommendations.

Although we recognize that treatments for other *KRAS* mutations are under investigation, they are beyond the scope of these recommendations. The focus herein is on *KRAS G12C* because it is currently the only *KRAS* mutation with available treatments supported by phase 3 trial evidence and an indication from Health Canada.

The strengths of individual recommendations are based on the level of supporting evidence and indicated by the verbs “should”, “could”, and “may”, where “should” indicates the highest level of evidence supported by RCTs; “could” indicates alternative options supported by a lower level of evidence (i.e., non-randomized trials); and “may” indicates expert opinion.

## 2. KRAS Mutations: Biology and Signaling Pathways

*KRAS* belongs to the *RAS* superfamily that encodes the signalling proteins responsible for the mitogen-activated protein kinase (MAPK) and phosphatidylinositol 3 kinase (PI3K) pathways. Downstream signalling is controlled by switching between the active state (guanosine triphosphate [GTP]) and the inactive state (guanosine diphosphate [GDP]) [7,8]. This group of genes is involved in the control of cell growth, survival, differentiation and potentially shaping the local tumour-immune microenvironment [9,10], and is frequently associated with genomic alterations in human cancers. *KRAS* is the most commonly mutated *RAS* isoform, making up approximately 85% of oncogenic *RAS* mutations in all cancer types [11]. *KRAS* mutations are seen in 61–86% of pancreatic ductal adenocarcinomas, 33–52% of colorectal adenocarcinomas, and 17–32% of lung adenocarcinomas. [10,12,13]

The *KRAS* proto-oncogene encodes an intracellular guanine nucleotide-binding protein (G protein) that belongs to the family of small GTPases. Its structure consists of six beta chains and five alpha helices encompassing a catalytic G domain and a C-terminal hypervariable region. The C-terminal hypervariable region is responsible for anchoring *KRAS* to the membrane, and the G domain binds guanine nucleotides to activate signalling [8,14].

**Figure 1:**
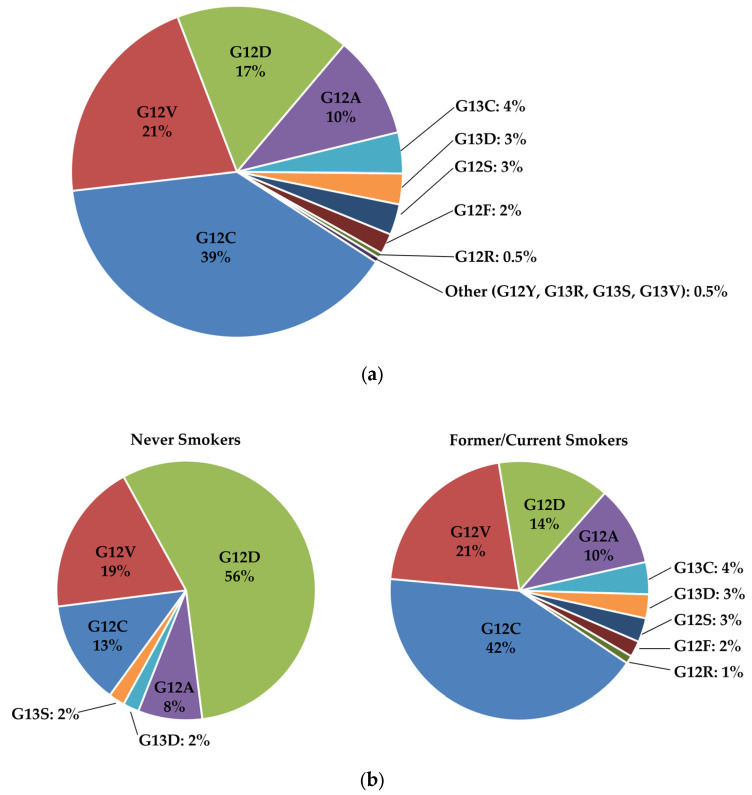
*KRAS* point mutations in codons 12 and 13 in patients with metastatic lung adenocarcinomas: (**a**) *KRAS* point mutations in all patients; (**b**) *KRAS* point mutations separated by smoking status. Reused and adapted with permission from Elsevier [17]. (**b**) In addition, there are reports of *KRAS* mutations outside codons 12 and 13, including codon 61 (i.e., Q61H) and 146 with a rate of <1% [15].

*KRAS* mutations are typically point missense mutations that frequently occur in codons 12 (89%), 13 (9%), and 61 (1%) [15] of the G domain to impair its GTP hydrolysis capacity and lead to activation of *KRAS* proteins and promotion of the GTP-bound active state [7,16]. The pattern of mutations of codons and substitutions of amino acids varies among tumour types. The most frequent mutations in *KRAS*-mutated NSCLC are transversion mutations involving nucleotide changes at codon 12 from guanine to uracil resulting in G12C and accounting for 40–50% of all *KRAS* mutations, followed by a glycine- to-valine substitution (G12V) accounting for approximately 25% of *KRAS* mutations, Figure 1A [17,18]. While transversion mutations leading to G12C and G12V are associated with a history of tobacco use, transitions mutations involving changes from guanine to adenine and resulting in a substitution of glycine to aspartic acid (G12D, 6–17% of all *KRAS* mutations) are found mainly in patients without exposure to tobacco smoke, Figure 1B [16,17]. *KRAS G12D* mutation is observed in approximately 35–45% of colorectal adenocarcinomas and 45–49% of pancreatic ductal adenocarcinomas [10,12].

*KRAS* mutations are often mutually exclusive of other oncogenic drivers [19,20,21]. However, rare cases (<1%) harbouring mutations in both *KRAS* and drivers of lung cancer development, such as *EGFR*, anaplastic lymphoma kinase (*ALK*) and *ROS*1, have been reported [22,23]. Co-occurring mutations in non-oncogenic drivers are relatively common, occurring in over half of patients with *KRAS* mutations [24,25]. The most frequent and functionally significant co-occurring mutations include tumour protein p53 (*TP53;* 42%), serine/threonine kinase 11 (*STK11*; 29%), kelch-like ECH-associated protein 1 (*KEAP1*; 27%) [25]. The *STK11* gene encodes the liver kinase B1 (LKB1) protein, the commonly altered tumour suppressor in NSCLC [26,27]. KEAP1 is a negative regulator of nuclear factor, erythroid 2 like 2 gene (NFE2L2), a transcription factor that binds to antioxidant response elements on the DNA and initiates the transcription of a number of genes involved in regulation of redox balance and cellular detoxification [28].

These genomic alterations likely co-occur early during oncogenesis [24,25,29] and, along with *KRAS* mutations, may drive oncogenesis and influence therapeutic responses, as well as the ability of tumour cells to acquire resistance [30,31,32]. Thus, co-occurring mutations have the potential to alter prognosis, with *STK11* and *KEAP1* mutations being associated with a shorter duration of response to initial chemotherapy and shorter overall survival from initiation of immune checkpoint inhibitors (ICIs), making co-mutation of *KRAS* and *KEAP1/NFE2L2* an independent prognostic factor [25,33,34]. KRAS with concomitant *STK11/LKB1* loss has also been associated with a tolerogenic tumour-immune microenvironment with increased immunosuppressive myeloid cell infiltration, increased T cell exhaustion, decreased tumour PDL1 expression, and a reduced likelihood of response to PD1 inhibitors [35].

## 3. Epidemiology, Clinical Features, and Prognostic Implications

### 3.1. Overview

The frequency of *KRAS* mutations in patients with lung cancer varies from 20% to 40% in adenocarcinomas and approximately 2–4% in squamous cell carcinomas [5,36]. According to unpublished data from British Columbia (2042 samples), *KRAS* mutations were present in 33% of patients with stage IIIb/IV NSCLC, and the frequency of *KRAS G12C* was 13%. *KRAS* mutations are more frequent in Caucasian and Hispanic (25% to 35%) than in Asian populations (9% to 24%) and are more frequent in patients with a history of tobacco use (30%) than in those without exposure to tobacco smoke (10%) [37,38,39,40,41,42]. Tobacco use is more common in rural areas and among people with less education and lower incomes [43,44]. Thus, the negative impact on cancer outcomes associated with geography and lower socioeconomic status, including reduced access to healthcare, more frequent late-stage diagnosis, limited treatment options, and ultimately a higher risk of disease progression and early mortality is disproportionately felt among groups with tobacco-associated *KRAS* mutations. Finally, the ability to access oral take-home cancer medications may be limited for patients not living in jurisdictions where this class of medication is covered as part of publicly funded oncology care. Even for those with private drug insurance, the co-pays may exceed a patient’s ability to afford a KRAS-targeting medication [45]. Awareness of the socioeconomic and geographic disparity is critical for healthcare teams to provide the best care, which aims to address the current inequities in access to direct care, including access to biomarker testing and the best systemic therapies.

Attempts have been made to define the predictive value of KRAS mutations in NSCLC patients in response to standard chemotherapy [46,47], targeted therapies [48,49] and ICIs [50]. Preclinical data from Garassino et al. [51] suggested that the *KRAS* mutation subtype should be considered when investigating its potential role as a predictive biomarker for chemotherapy in NSCLC. For example, while *KRAS G12C* clones were associated with a reduced response to cisplatin and an increased sensitivity to paclitaxel and pemetrexed, the *KRAS G12D* variant led to resistance to paclitaxel treatment. Furthermore, *KRAS G12V* clones were associated with a better response to cisplatin compared to *KRAS* wild type. *KRAS G12V* clones also appeared to be more resistant to pemetrexed. Sun et al. reported significantly poorer survival in patients with *KRAS* mutations treated with pemetrexed or gemcitabine but not in those receiving taxanes [46].

Programmed death ligand 1 (PD-L1) expression has been shown to correlate with KRAS status, and *KRAS* mutations were described as possible biomarkers for ICI efficacy. A large international retrospective study (IMMUNOTARGET) assessed the benefits of ICIs in 551 patients with advanced NSCLC and oncogenic driver alterations, including 271 patients with *KRAS* mutations [52]. The study demonstrated a higher objective response rate (ORR) with ICIs in patients with *KRAS* mutations (26%) compared to patients with other oncogene drivers. Progression-free survival (PFS) and response rates were not significantly different within *KRAS* mutation subtypes and patients with KRAS-mutated non-squamous NSCLC treated with ICIs had better outcomes than those with wild-type KRAS tumours [53]. This may be explained by the higher overall level of PD-L1 expression in KRAS-mutated tumours [54]. In a study by Lauko et al., patients with KRAS-mutated NSCLC and brain metastases had higher responses to ICIs compared to those without KRAS mutations [55]. However, it should be noted that some KRAS mutations are associated with smoking and, therefore, have increased tumour mutation burden (TMB)—a biomarker associated with the increased efficacy of ICIs [56,57].

### 3.2. Co-Mutations and Their Impact on Treatment Outcomes

Prognosis associated with *KRAS* mutations may depend on co-alterations. Several retrospective analyses suggest poor survival outcomes in patients with *KRAS* mutation co-occurring with *STK11* or *KEAP1* treated with ICIs, regardless of PD-L1 expression [20,24,58,59]. According to a report from four US cancer centres, the *STK11* and *KEAP1* mutations confer worse outcomes with ICIs in patients with KRAS-mutated NSCLC but not those with wild type KRAS NSCLC. *KRAS* mutations co-occurring with either STK11 or KEAP1 mutations are associated with significantly worse progression-free (STK11 hazard ratio [HR] = 2.04, *p* < 0.0001; KEAP1 HR = 2.05, *p* < 0.0001) and overall (STK11 HR = 2.09, *p* < 0.0001; KEAP1 HR = 2.24, *p* < 0.0001) survival when treated with ICIs [59]. The report was based on 1261 patients with lung adenocarcinoma, out of which 536 (42.5%), 260 (20.6%), and 231 (19.2%) harboured the *KRAS*, *STK11* and *KEAP1* mutations, respectively. Further analyses revealed that the presence of *STK11* or *KEAP1* mutations result in distinct immunophenotypes in KRAS-mutated, but not in KRAS-wild type NSCLC.

Evidence suggests that KRAS/STK11 and KRAS/KEAP1 co-alterations may be prognostic rather than predictive and may also be associated with poor outcomes not only with ICIs but when treated with chemotherapy and targeted therapy [60,61]. It is important to note that conclusions should not be made due to the retrospective nature of the available studies, the small subgroups of patients tested, and the heterogeneous testing of other co-occurring mutations. Moreover, the studies did not include comparative arms and raise the question of whether similar outcomes could be achieved regardless of the treatment. In light of current evidence, the presence of STK11 and KEAP1 mutations in patients with KRAS G12C mutation should not discourage the administration of an ICI, either as a single agent or in combination with platinum-based chemotherapy. Further prospective studies with appropriate controls are needed to clarify whether co-occurrence of STK11 and KEAP1 and their association with KRAS mutation is an independent poor prognostic factor or if they are associated only with poor outcomes with ICIs.

## 4. Identifying Patients with KRAS G12C

### 4.1. Testing Strategies

*KRAS* mutations, in particular, *KRAS G12C*, can be detected with tissue (biopsy or surgically resected sample), various cytology samples, or blood [62]. The addition of *KRAS G12C* to the list of molecular alterations that should be tested for in NSCLC requires molecular pathology laboratories to amend their diagnostic algorithms and re-consider the quality and quantity of the DNA, as well as the turnaround time (TAT) to send the report to medical oncologists. *KRAS* mutations can be detected with either a comprehensive panel or targeted testing. Considering the increasing number of biomarkers of interest for targeted therapies for patients with NSCLC as well as other cancers (i.e., KRAS testing in colorectal cancer), it is easier, more cost-effective, and less tissue-consuming to test for KRAS with a next generation sequencing (NGS) approach with a large multigene panel than by a single gene test. The identification of KRAS mutations with NGS requires 10–15 ng DNA for amplicon-based or 40–50 ng DNA for hybrid capture technologies [63]. Large panels (more than 300 genes) require more DNA than medium-sized panels (from 20 to 50 genes).

For treatment decision making, it is required to assess and report on PD-L1 expression. Other relevant co-alterations (e.g., *TP53*, *STK11*, and *KEAP1*) could be evaluated concurrently. However, the panel recognizes that currently, not all centres have these co-alterations included in their NGS panels. Furthermore, the predictive role of co-occurring mutations needs further assessment in prospective clinical trials before recommendations regarding the inclusion of *STK11* and *KEAP1* into NGS panels and routine testing could be made. Thus, although we cannot make a recommendation regarding testing for these co-alterations, we strongly encourage centres and molecular laboratories to consider including *STK11* and *KEAP1* in their panels. Retrospective data are compelling, and these biomarkers are likely to become relevant for therapeutic decision making in the near future.

### 4.2. Testing for KRAS G12C

Testing for *EGFR* mutations at diagnosis with NGS is approved in Canada for all non-squamous NSCLC stage IB to IV. Reflex testing for *KRAS* could be concurrently initiated by the pathologist. Additional testing for *KRAS G12C* could also be considered at the time of disease progression or recurrence if not completed previously.

Accessibility to NGS in routine practice, and thus assessment of KRAS status, varies across Canada. As such, medical oncologists should be aware of the techniques used by the molecular laboratory at their centre, as well as limitations of their testing methods.

### 4.3. Should Liquid Biopsy Be Integrated into KRAS Testing in Patients with NSCLC?

Tissue biopsy is currently the gold standard for molecular analysis in NSCLC, and it is valuable for determining the type of cancer, tumour gene expression, and the presence of mutations. However, it is affected by several factors related to tumour origin, including tumour location, accessibility for surgical removal, and available tissue.

Liquid biopsy may be used as a complementary or alternative approach to tissue-based genomic testing. It is a less invasive procedure with a faster TAT. Since it analyzes cell-free DNA (cfDNA) from cells shed into the bloodstream as opposed to being limited to the DNA from a single biopsied lesion, liquid biopsy may provide a more comprehensive overview of all the mutations found in different lesions, including metastases and secondary tumours. Thus, liquid biopsy offers potential advantages over tissue biopsy for molecular profiling because it may overcome identification issues related to tumour heterogeneity [64].

Liquid biopsy also has limitations. For advanced NSCLC, studies have documented the presence of circulating tumour DNA (ctDNA) in about 85% of cases, highlighting that a negative liquid biopsy result does not necessarily indicate the absence of a specific mutation [65]. Thus, although driver mutations found by liquid biopsy should be considered actionable, negative results must be considered inconclusive and, if possible, be confirmed by tissue biopsy [66].

When interpreting the presence of *KRAS* mutations in tumour cells, one must distinguish them from *KRAS* mutations associated with clonal hematopoiesis of indeterminate potential (CHIP), which occurs in older adults and presents pitfalls for liquid biopsy assessment [67,68,69]. *KRAS* mutations can also be identified in certain non-malignant diseases and, theoretically, can be found when using very sensitive ctDNA approaches [70,71,72,73]. In addition, *KRAS* mutation found by liquid biopsy might not necessarily indicate the origin of the tumour (i.e., lung) but rather lung metastases from a tumour originating elsewhere (i.e., pancreas). It is also incorrect to assume that a primary tumour will release as many cells into the bloodstream as metastases present in the same patient and that all mutations can be expected to be represented by ctDNA equally well. Although liquid biopsy can potentially find mutations from different sites, it may not detect mutations from tumours that do not shed enough cells into the bloodstream or mutations found only in a small area of tumour.

Despite the described limitations, numerous studies have demonstrated a relatively good correlation between liquid and tissue biopsy results [74]. Thus, liquid biopsy is a promising tool to systematically detect different actionable genomic alterations at diagnosis and at tumour progression or recurrence, notably in patients treated with different anti-EGFR or anti-ALK TKIs [66]. In the absence of tissue material, *KRAS* status, notably *KRAS G12C*, can be evaluated using liquid biopsy [75]. Both blood and fluid samples (i.e., bronchial aspirates, bronchoalveolar lavage, pleural and cerebrospinal fluid) can be used for liquid biopsy [62]; however, different sampling techniques will yield different amounts of DNA. As such, multi-centric and independent validation studies are needed to establish the reproducibility and the robustness of different liquid biopsy techniques, as well as to determine the specific steps of pre-analytical phases. It is essential that laboratories thoroughly validate all aspects of DNA extraction and sequencing, especially the initial amount of DNA and average sequencing depth [76,77].

### 4.4. TAT and Reporting of Biomarker Test Results

Rapid TAT for biomarker test results, especially in newly diagnosed NSCLC requiring therapy, is necessary for timely treatment initiation. The College of American Pathologists recommends a maximum 10-day TAT from sample receipt in the laboratory to report generation [78]. It is also recommended for pre-laboratory TAT to not exceed 3 business days and post-laboratory TAT to be less than 24 h [79]. However, the maximum acceptable time to wait for biomarker results for each patient should be at the clinician’s discretion and balanced between missed treatment opportunities and the benefits of waiting for appropriate targeted therapy.

Reporting KRAS mutations, specifically *KRAS G12C*, can vary across tumour types. In NSCLC, specific *KRAS* mutations should be reported at the variant level [80]. Although there are various ways that *KRAS G12C* can be reported (i.e., KRAS p.Gly12Cys or KRAS c.34G > T), pathologists should consider noting *KRAS G12C* in the report synopsis for clarity and ease of interpretation [81].

### 4.5. Recommendations

- Testing for *KRAS G12C* should be performed as part of a comprehensive panel that includes current standard-of-care biomarkers as summarized by international guidelines. Single-gene strategies for KRAS are not recommended.
- Although KRAS G12C status is currently required for patients with stage III/IV NSCLC, KRAS G12C could be included in the NGS panel and reflex biomarker testing for *KRAS G12C* could be initiated by the pathologist at the time of initial diagnosis in all patients diagnosed with stage IB-IV non-squamous NSCLC, if NGS is performed.
- The panel recognizes the lower frequency of *KRAS G12C* and other actionable alterations in squamous cell carcinoma and, in light of current evidence, suggests broad molecular testing in patients with advanced disease in which systemic therapy is being considered if resources are available and there is no significant additional burden on the molecular pathology laboratory.
- *TP53, STK11*, and *KEAP1* are evolving biomarkers that may have implications in patients with *KRAS* mutations; therefore, molecular laboratories could consider preparing to include these alterations in NGS panels as they may become relevant for therapeutic decision making in the near future as more supporting data becomes available.
- Liquid biopsy should be considered when tissue biopsy is unavailable or inadequate for molecular testing, when invasive procedures for tissue procurement are contraindicated, or when urgent treatment decisions are required and delays are expected with tissue testing.
- Liquid biopsy results should be interpreted by qualified professionals in a patient-specific context. Providers should be aware of the limitations and use caution when interpreting standalone liquid biopsy results, as liquid biopsy is not a diagnosis in and of itself but rather augments our understanding of a histologically or cytologically diagnosed malignancy.
- Negative liquid biopsy results do not mean the absence of the target and tissue testing is recommended if liquid biopsy does not detect a mutation indicating the presence of tumour-derived DNA.
- The maximum acceptable TAT (from the acquisition of tissue to the oncologist having the report) for all biomarkers should not exceed 21 calendar days. Accelerated testing should be available for certain specific situations.
- Biomarker test results should be compiled and ideally reported in a single comprehensive biomarker report by the pathologist that includes information on PD-L1 expression.

## 5. Therapeutic Approaches for Patients with Advanced NSCLC Harbouring KRAS Mutations

### 5.1. Questions

- What is the preferred first-line therapy for treatment-naïve patients with KRAS mutations?
- What are the preferred subsequent lines of therapy for patients with KRAS mutations?

### 5.2. ICIs in Patients with KRAS Mutations

Currently, for patients with advanced NSCLC without driver alterations in *EGFR* and *ALK*, the American Society of Clinical Oncology (ASCO) and Ontario Health (Cancer Care Ontario [CCO]) Expert Panel recommends monotherapy with an ICI (for patients with high PD-L1 expression; tumour proportion score [TPS] ≥ 50%) or an ICI in combination with chemotherapy (for patients irrespective of PDL1 expression) [82].

The ASCO/OH (CCO) recommendations are based on the KEYNOTE-024 [83], KEYNOTE-189 [84], and KEYNOTE-407 [85] trials that demonstrate favourable long-term outcome effects with ICI monotherapy in patients with a TPS or PD-L1 of ≥50%, as well as with an ICI in combination with chemotherapy. Subsequently, the KEYNOTE-042 trial confirmed that ICI monotherapy can be extended as first-line therapy to patients with locally advanced or metastatic NSCLC with low PD-L1 TPS [86].

In the Checkmate 227 trial, first-line nivolumab plus ipilimumab improved OS compared to chemotherapy in patients with NSCLC, independent of PD-L1 expression level [87], and the Checkmate 9LA trial confirmed that nivolumab plus ipilimumab with two cycles of chemotherapy provided a significant improvement in OS versus chemotherapy alone [88]. The IMpower110 (NCT02409342) trial also demonstrated a statistically significant improvement in OS in patients with high PD-L1 tumour expression (PD-L1 stained ≥ 50% of tumour cells [TC ≥ 50%] or PD-L1 stained tumour-infiltrating immune cells [IC] covering ≥ 10% of the tumour area [IC ≥ 10%]) receiving atezolizumab compared to those treated with platinum-based chemotherapy [89]. There was no statistically significant difference in OS for the other two PD-L1 subgroups (TC ≥ 5% or IC ≥ 5%; and TC ≥ 1% or IC ≥ 1%) at the interim or final analyses. The EMPOWER-Lung 3 trial confirmed the efficacy of cemiplimab, another anti-PD-1/PD-L1 agent, in advanced NSCLC as both monotherapy and in combination with chemotherapy in both squamous and non-squamous histologies [90].

All of the trials included patients with KRAS mutations. An exploratory analysis of KEYNOTE-042 showed that pembrolizumab monotherapy was associated with a 58% reduced risk of death (HR, 0.42 [95% CI, 0.22–0.81]) in patients with any KRAS mutation and a 72% (HR, 0.28 [95% CI, 0.09–0.86]) reduced risk in patients with KRAS G12C [91]. The benefit from an ICI was limited by concomitant *STK11/LKB1* mutations. Similarly, the exploratory analysis of KEYNOTE-189 trial suggested benefits of first line ICI plus chemotherapy in patients with metastatic non-squamous NSCLC regardless of KRAS mutation status. [92] In both KEYNOTE-042 and KEYNOTE-189, patients with KRAS mutations had higher TMB compared to those without KRAS mutation (191 vs. 105 mut/exome in KEYNOTE 042; 204 vs. 141 mut/exome in KEYNOTE-189) [91,92].

To better define the benefit of first-line ICIs with or without chemotherapy according to KRAS mutational status and PD-L1 expression in patients with advanced NSCLC, the Food and Drug Administration (FDA) conducted a pooled analysis using data from 12 registrational clinical trials. KRAS mutational status was reported in 1430 patients (61% unmutated, 39% mutated) [93]. The analysis confirmed that patients with KRAS-mutated NSCLC benefit from first-line ICI plus chemotherapy similarly to those with KRAS wild-type NSCLC and should receive upfront combination therapy. Patients with KRAS-mutated NSCLC derived the greatest benefit from the combination when compared to an ICI or chemotherapy alone. The small number of patients with documented KRAS G12C limits the interpretation of the data for this subgroup. It is important to note that the KRAS mutations group did not account for co-mutations (i.e., *STK11*) and the optimal therapy (ICI + chemotherapy or ICI monotherapy) in patients with PD-L1 ≥ 50% is not determined.

**Figure 2:**
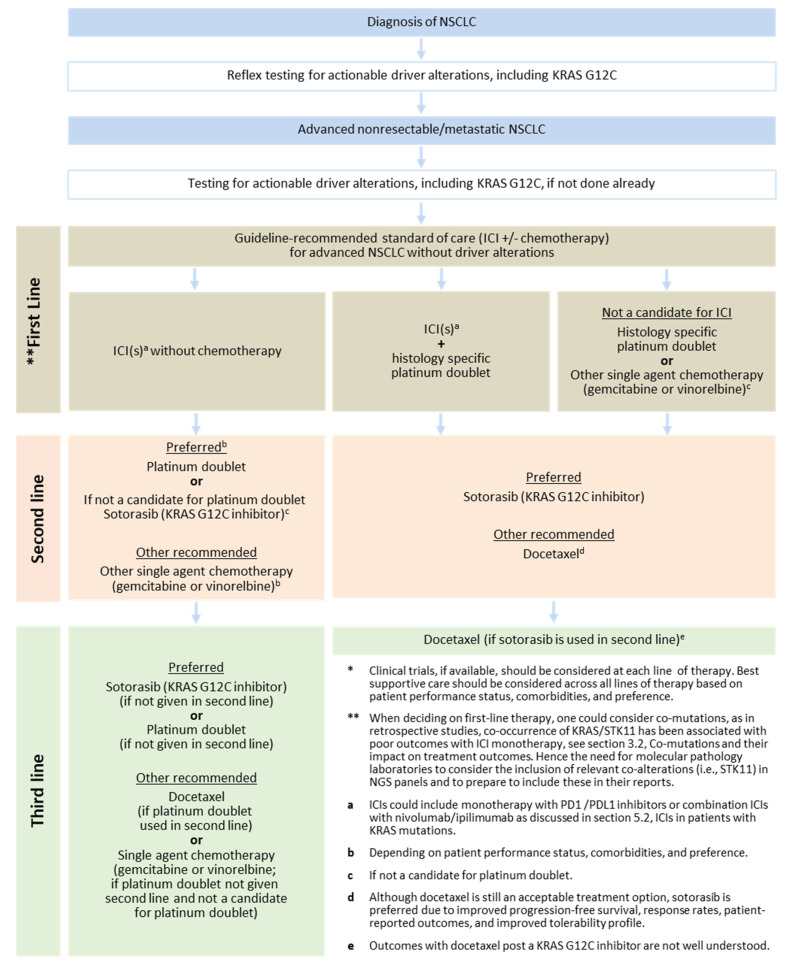
Canadian Treatment Algorithm for KRAS G12C-mutated NSCLC*.

With the availability of novel KRAS-targeted therapy, the sequencing of therapies in KRAS-mutated NSCLC is becoming increasingly complex. Figure 2 provides the algorithm proposed by our group for the management of *KRAS G12C* mutated NSCLC. The algorithm takes into consideration the ASCO/OH (CCO) recommendations and recent evidence that supports the use of KRAS G12C inhibitors.

### 5.3. Recommendations: First-Line Treatment of Advanced KRAS G12C-Mutated NSCLC

- Patients with advanced *KRAS G12C*-mutated NSCLC that are eligible for treatment should be offered guideline-recommended standard of care for advanced NSCLC without driver alterations (ICI(s) +/− chemotherapy), Figure 2.
- The impact of co-mutations, including *TP53*, *STK11*, and *KEAP1*, is currently under investigation and the presence of these mutations should not impact first-line treatment decisions at present.

### 5.4. Direct Inhibition of KRAS

#### 5.4.1. Overview

Recently, two molecules directly targeting KRAS G12C, sotorasib and adagrasib, completed phase 2 (NCT03600883, NCT03785249) and phase 3 trials (NCT04303780, NCT04685135) in patients with previously treated NSCLC, are shown in Table 1. Their mechanisms of action are based on the conversion of preference of KRAS from GTP to GDP, holding a KRAS state from an active to inactive GDP-bound form, and interrupting intracellular signaling and tumour cell growth.

#### 5.4.2. Sotorasib

In the Phase 2 CodeBreak100 trial (NCT03600883), treatment with sotorasib (960 mg daily) led to an ORR of 37%, median PFS of 6.8 months, and a median overall survival (OS) of 12.5 months, Table 1 [4]. The trial included 126 patients with locally advanced or metastatic KRAS G12C-mutated NSCLC who had progressed on prior standard therapies (81.0% progressed while on a combination of ICI and platinum-based chemotherapy). As for histologic subtypes, 95% had adenocarcinoma, 2% large-cell carcinoma and 1% squamous cell carcinoma. Exploratory analyses demonstrated the clinical activity of sotorasib across a range of co-occurring mutation profiles (i.e., KEAP1 and/or STK11 co-occurring mutations). Treatment-related adverse events (TRAEs) were reported in 70% of patients. The most common ones were diarrhea (31.7%), nausea (19.0%), and increases in alanine aminotransferase (ALT; 15.1%) and aspartate aminotransferase (AST; 15.1%), Table 2.

Efficacy and safety data from the CodeBreaK100 trial supported the FDA and Health Canada approvals of sotorasib for the treatment of patients with KRAS-mutant NSCLC. In Canada, sotorasib is approved for the treatment of adult patients with KRAS G12C-mutated locally advanced (not amenable to curative therapy) or metastatic NSCLC who have received at least one prior systemic therapy [3].

The CodeBreaK200 trial, is a global, randomized phase 3 trial in NSCLC, comparing sotorasib (960 mg daily) versus docetaxel (75 mg/m^2^ IV Q3W) in second line and beyond (NCT04303780) [5]. The trial included 345 patients with at least one prior treatment, including platinum-based chemotherapy and ICI. Treatment with chemotherapy and ICI could be concurrent or sequential, and patients with medical contraindications to these therapies could be included with approval. After a median follow-up of 17.7 months, the median PFS (primary endpoint) was 5.6 months in the sotorasib group and 4.5 months in the docetaxel group, giving a statistically significant hazard ratio (HR) of 0.66 in favour of sotorasib, Table 1. At 1 year, the PFS rate was 24.8% with sotorasib and 10.1% with docetaxel. The ORR was also significantly higher with sotorasib than with docetaxel, at 28.1% vs. 10.1% (*p* < 0.001). Furthermore, patients in the sotorasib arm had a faster median time to response than those in the docetaxel arm (1.4 vs. 2.8 months) and a longer median duration of response (DoR; 8.6 vs. 6.8 months). Time to deterioration in global health status, physical functioning, and cancer-related symptoms (dyspnoea and cough) were delayed with sotorasib compared to docetaxel. OS did not differ significantly between the two treatment arms, at a median of 10.6 and 11.3 months with sotorasib and docetaxel, respectively. However, it has been noted that due to the FDA’s guidance to reduce the size of the trial by half when positive data from the single-arm CodeBreaK100 study became available, the CodeBreaK200 trial was not adequately powered to detect OS differences. The FDA also asked for the study protocol to be changed to allow for treatment crossover and 34% of patients in the docetaxel arm subsequently crossed over and received subsequent KRAS G12C inhibitor, including 46 (26%) who crossed over to sotorasib as per the study protocol and 13 (7%) who received a KRAS G12C inhibitor as a subsequent therapy following discontinuation from study treatment. 62 (36%) patients treated with sotorasib received subsequent therapy; 36 (21%) patients were known to have received subsequent chemotherapy.

#### 5.4.3. Adagrasib

Adagrasib (MRTX849) is another oral covalent KRAS G12C inhibitor that irreversibly and selectively binds KRAS G12C. It was evaluated in a phase 1/2 study (KRYSTAL-1; NCT03785249) [94]. The study enrolled 116 patients (113 [97.4%] with adenocarcinoma and 3 [2.6%] with squamous cell carcinoma) with histologically confirmed unresectable or metastatic NSCLC, 98.3% of whom had previously received both chemotherapy and immunotherapy. Of 112 patients with measurable disease at baseline, 48 (42.9%) had a confirmed objective response. The ORRs were similar across PD-L1 expression subgroups, indicating that the efficacy of adagrasib was not affected by PD-L1 expression. The ORRs in patients with co-mutations in STK11, KEAP1, TP53, and CDKN2A range from 28.6% to 58.3%. The median DoR was 8.5 months (95% CI, 6.2 to 13.8), and the median PFS was 6.5 months (95% CI, 4.7 to 8.4). As of 15 January 2022 (median follow-up, 15.6 months), the median OS was 12.6 months (95% CI, 9.2 to 19.2). 97.4% of the patients experienced TRAEs, most of which were grade 1 or 2, Table 2. A total of 52 patients (44.8%) had grade 3 or higher TRAEs. Two grade 5 fatal events were reported (one cardiac failure in a patient with a medical history of pericardial effusion and one pulmonary hemorrhage). TRAEs led to dose reduction in 60 patients (51.7%), dose interruption in 71 patients (61.2%) and treatment discontinuation in 6.9% of patients.

Based on the data from the KRYSTAL-1 trial, in December 2022, the FDA granted accelerated approval to adagrasib for adult patients with *KRAS G12C*-mutated locally advanced or metastatic NSCLC, as determined by an FDA-approved test, who have received at least one prior systemic therapy [95]. At the time of this publication, adagrasib was not approved by Health Canada; thus, it is not included in the treatment algorithms and recommendations for Canadians. If adagrasib or any other KRAS inhibitor currently in development receives the Health Canada indication, the amendments could be made accordingly.

Sotorasib and adagrasib have recently been added to the National Comprehensive Cancer Network (NSCLC) guidelines as a subsequent therapy option for patients with KRAS G12C mutated NSCLC that progressed after at least one line of therapy, if no previous KRAS G12C-targeted therapy was given. Because adagrasib and sotorasib have a similar mechanism of action, it is not recommended to switch between these agents at the time of progression [96]. See Figure 2.

Other KRAS G12C inhibitors in development include GDC-6036, JDQ-443, and MK-1084. According to preclinical studies and in vitro data, GDC-6036 is presumed to be more potent and selective than sotorasib and adagrasib [97]. JDQ443 is designed with a novel binding mechanism [98] and MK-1084 is being investigated in phase 1 trial as a monotherapy and in combination with pembrolizumab in patients with KRAS G12C mutant advanced solid tumours (MK-1084-001; NCT05067283).

According to data from a phase 1a trial (NCT04449874) presented at the 2022 World Conference on Lung Cancer, GDC-6036 elicited an ORR of 53% in efficacy-evaluable patients with NSCLC harboring KRAS G12C (*n* = 57) [99]. All 30 responders experienced partial responses. Similar to other KRAS G12C inhibitors, the most common grade ≥ 3 TRAEs included increased ALT (6.8%), increased AST (5.1%), diarrhea (3.4%), and fatigue (1.7%). No dose-limiting toxicities were reported. AEs leading to GDC-6036 dose modifications, reductions, or withdrawal were reported in 36%, 19%, and 5% of patients, respectively.

The dose-escalation portion of the phase 1b/2 KontRASt-01 trial (NCT04699188) evaluating JDQ443 in patients with KRAS G12C-mutant solid tumours was presented at the 2022 American Association for Cancer Research (AACR) Annual Meeting [100]. Among the subgroup of patients with NSCLC, the ORR was 45%, with confirmed partial responses reported for seven patients (35%). Investigators observed stable disease in 55% of patients, with no reports of progressive disease and two unevaluable patients in the cohort. The confirmed ORR at the recommended phase 2 dose (200 mg twice daily) was 57%.

### 5.5. Recommendations: Second- and Subsequent-Line Treatment of Advanced KRASs G12C-Mutated NSCLC

- For patients with locally advanced or metastatic KRAS G12C-mutated NSCLC who have progressed on ICI(s) and/or chemotherapy, the panel recommends sotorasib over docetaxel based on improved progression-free survival, response rates, patient-reported outcomes, and improved tolerability profile.
- It is reasonable to offer sotorasib as second-line therapy to patients who progressed on only having either monotherapy ICI that are not candidates for platinum doublet or patients with a contraindication to ICIs that received chemotherapy in the first-line setting
- Given that sotorasib is approved by Health Canada and has phase 3 trial data, the panel recommends the use of sotorasib in the current treatment algorithm of advanced KRAS G12C mutated NSCLC. However, the panel recognizes that the use of adagrasib or a clinical trial with another KRAS G12C inhibitor (monotherapy or in combination) is an acceptable treatment option in this line of therapy.
- Sotorasib and adagrasib have a similar mechanism of action; it is not recommended to switch between these agents at the time of progression.

### 5.6. What Are the Treatment Options for Metastatic NSCLC Patients with KRAS G12C and Brain Metastases?

#### 5.6.1. Overview

Brain metastases develop in up to 40% of patients with stage IV NSCLC [101], and the incidence among NSCLC patients with KRAS mutations is similar. The activity of therapy against brain metastases is essential for maintaining disease control and quality of life. An open-label, phase 2 study indicated the efficacy of ICIs in PD-L1 positive (PD-L1 expression ≥ 1%) stage IV NSCLC with brain metastasis, with 29.7% of patients achieving a response [102]. Of the patients, 93% were former or current tobacco users, and 53% had driver mutations (14 patients had KRAS mutations).

Limited preliminary data indicate that both adagrasib and sotorasib penetrate the blood–brain barrier and have activity in brain metastases [94,103]. In the KRYSTAL-1 trial, 42 patients had brain metastases at baseline [94], 82% of which received radiation therapy before adagrasib treatment (59% within 3 months of study entry, and 37% at least 6 months before study entry). Of the 33 patients who could be evaluated radiographically, the confirmed intracranial ORR was 33.3% (95% CI, 18.0 to 51.8), and the median duration of intracranial response was 11.2 months (95% CI, 2.99 to not evaluable). The median intracranial PFS in patients with baseline brain metastases was 5.4 months (95% CI, 3.3 to 11.6).

Although patients with active brain metastases were excluded from the CodeBreaK100 trial, those with stable, asymptomatic brain metastases were included. Out of 40 patients with baseline brain metastases, 65% (*n* = 26) received prior radiation therapy, 20% (*n* = 8) underwent surgery, and 12% (*n* = 5) had both radiation therapy and surgery. At a median follow-up of 12 months, sotorasib led to a confirmed ORR of 25% in patients with baseline brain metastases compared with 42% in patients without baseline brain metastases. The disease control rates (DCRs) were 77.5% vs. 84.1%, respectively [103]. Among 16 patients with evaluable brain metastases, the intracranial disease control rate was 88% (*n* = 14).

The CodeBreak200 trial also excluded patients with new or progressing untreated or symptomatic brain lesions. Patients with treated, stable brain metastases were eligible. Brain metastases at baseline were more prevalent in CodeBreaK 200 than in CodeBreaK 100 (34% vs. 23%). In a pre-planned exploratory analysis of patients with previous CNS disease in the CodeBreak200 trial, the median time to recurrence of CNS disease, as per investigator assessment, was delayed with sotorasib compared with docetaxel (15.8 months [95% CI 9.7–not estimable] vs. 10.5 months [5.8–not estimable]; HR 0.52 [95% CI 0.26–1.0]).

Several trials evaluating sotorasib in patients with brain metastases are planned or already recruiting. The ongoing phase 1/2 CodeBreaK101 trial (NCT04185883) is evaluating sotorasib monotherapy and sotorasib in combination with other anticancer therapies in patients with KRAS G12C–mutated advanced solid tumours and active untreated brain metastases. A phase 1/2 trial investigating sotorasib in combination with MVASI (a bevacizumab biosimilar) in patients with advanced KRAS G12C-mutant NSCLC with small, untreated brain metastases (NCT05180422) is underway. Results of these studies will provide more information on the optimal management of patients and intracranial activity of sotorasib.

#### 5.6.2. Recommendations

- KRAS G12C inhibitors, sotorasib and adagrasib, appear to have activity in brain metastases, but there is insufficient evidence to recommend one agent over the other in patients with brain metastases.
- Currently, there is insufficient evidence to make recommendations regarding the sequencing of KRAS G12C inhibitors and radiation in patients with brain metastases.
- Due to uncertainties related to potential interaction with radiation therapy, the panel does not recommend concurrent use of KRAS G12C inhibitors with stereotactic radiosurgery (SRS) or whole-brain radiation therapy (WBRT).

### 5.7. What Are the Common Toxicities Associated with KRAS G12C Inhibitors and How Should They Be Managed?

#### 5.7.1. Overview

The main toxicities reported with KRAS G12C inhibitors are grade 1–2 gastrointestinal events (diarrhea, vomiting, and nausea), reported in up to 60% of patients and increases in liver enzymes reported in 15–25% of patients (3–6% grade ≥ 3). Table 2 summarizes the common TRAEs reported in phase 2 trials with sotorasib and adagrasib [4,94]. When interpreting these data, one should consider the differences in study protocols and reporting requirements, and direct comparisons should be avoided. It should, however, be noted that discontinuation rates due to AEs are similar in both trials (~7%).

One of the main concerns with KRAS G12C inhibitors is the risk of hepatotoxicity that may lead to drug-induced liver injury and hepatitis. In a post hoc analysis of CodeBreak200 higher incidence of treatment-related adverse events of grade 3 or worse and hepatotoxicity events were found in patients treated with immunotherapy ≤ 2.6 months or less before initiation of sotorasib, compared with those treated > 2.6 months before treatment with sotorasib. Overall, the greater the time between previous immunotherapy and sotorasib, the lower the incidence of treatment-related adverse events of grade 3 or worse and hepatotoxicity events.

Chour et al. reported the incidence of liver toxicity in patients treated with sotorasib in French compassionate or expanded access programs and outside clinical trials [104]. Out of 102 patients, 48 (47%) received sequential sotorasib following an ICI. Grade 3 sotorasib-related AEs were significantly higher in patients previously treated with ICI (50% vs. 13% *p* < 0.05). The grade ≥ 3 sotorasib-related AE excess was mainly driven by grade 3 and 4 elevations in liver enzymes. Grade 3 hepatitis was found in 22 out of 102 patients (21.6%) in the total cohort and significantly more frequent in patients previously treated with ICI (33 vs. 11, respectively [*p* < 0.05]). Grade 3 sotorasib-related AEs rate was significantly higher in patients receiving the last ICI infusion less than 90 days before sotorasib initiation (22/44 patients [50%] vs. 9/51 patients [18%], *p* < 0.05). Grade 3 hepatitis rate was also significantly higher in patients receiving the last immunotherapy injection less than 90 days before sotorasib initiation (16/44 patients (36%) vs. 6/51 patients (12%), *p* < 0.05). Similarly, data with EGFR inhibitor osimertinib post-ICI revealed a higher risk of irAEs within 3 months of prior PD-(L)1 blockade (24% vs. 13% within 3–12 months) [105]. This data indicates the need for more frequent and thorough monitoring for irAEs (including liver function test), if a patient is initiated on targeted therapy, including KRAS-G12C inhibitor, within 3 months post-ICI.

The mechanism of hepatotoxicity observed with sotorasib and other KRAS G12C inhibitors appears distinct from the typical drug-induced liver injury associated with other targeted therapies. A distinct autoimmune hepatitis has been associated with KRAS G12C inhibitors and is likely driven by previous exposure to ICI as described above. Indeed, a published case report described severe hepatitis on sotorasib in the setting of sequential sotorasib following anti-PD1 with liver biopsy consistent with autoimmune hepatitis [106]. As such, treatment with high-dose steroids should be considered in cases of severe hepatoxicity in contrast to the usual treatment of drug-induced liver injury with other targeted therapies. Consideration of low-dose prednisone to prevent flare in patients upon rechallenge with sotorasib should also be considered where appropriate in addition to dose reduction.

The sotorasib product monograph recommends monitoring liver function tests (ALT, AST, and total bilirubin) prior to the start of therapy and every 3 weeks for the first 3 months, then once a month or as clinically indicated, with more frequent testing in patients who develop transaminase and/or bilirubin elevations (see Table 3 for guidelines regarding the management of hepatotoxicity on the sotorasib). Clinicians should be aware that drug interactions were observed when sotorasib was co-administered with an acid-reducing agent, a strong CYP3A4 inducer, a P-glycoprotein (P-gp) substrate, and CYP3A4 substrates.

With the approval of KRAS G12C inhibitors and their use in routine practice, as well as in ongoing clinical trials in combination with other therapies, it is likely that clinicians will gain a better understanding of AEs related to KRAS G12C treatment, in particular, how to effectively prevent (i.e., a washout period between ICI and initiation of KRAS inhibitors) and manage liver toxicity to avoid treatment discontinuations and ensure optimal therapeutic outcomes. The CodeBreaK 100 (NCT03600883) and 101 (NCT04185883) trials, for example, assess sotorasib at doses ranging from 120–960 mg/day in combination with pembrolizumab 200 mg or atezolizumab 1200 mg given every 3 weeks in advanced KRAS G12C-mutated NSCLC. In the lead-in cohorts, sotorasib was given as monotherapy for 21 or 42 days before ICI was started, while in the concurrent cohorts, the agents were given together from the beginning. Regardless of the ICI used, toxicity was worse with concurrent rather than lead-in sotorasib [107]. For example, treatment-related adverse events of grade 3 occurred in 74% of the 19 patients in the concurrent sotorasib–pembrolizumab cohort and in 53% of the 19 in the lead-in sotorasib–pembrolizumab cohort. The corresponding rates for the atezolizumab cohorts, each with 10 patients, were 50% and 30%. However, there was a trend towards fewer liver enzyme elevations with lower doses. The investigators pointed out that based on these results, future investigations will focus on sotorasib as lead-in therapy in combination with pembrolizumab as first-line therapy for patients with advanced KRAS G12C NSCLC to further assess and better understand the benefit–risk ratio.

#### 5.7.2. Recommendations

- As KRAS G12C inhibitors can cause hepatotoxicity, regular monitoring of liver function (ALT, AST, and total bilirubin) is recommended. Clinicians should follow the drug’s product monograph for dose reductions, interruptions, or permanent discontinuations.
- Corticosteroids are recommended in patients presenting with grade 2–4 hepatotoxicity.
- The expert panel agreed that a low dose of prednisone might be considered for preventing another occurrence of hepatotoxicity upon re-challenge with a KRAS inhibitor.
- Clinicians should be aware of an increased risk of AEs, in particular hepatotoxicity, when initiating sotorasib post-ICI.
- Sequencing of KRAS G12C inhibitors, ICI, and the need for and duration of washout period between these agents is yet to be determined. Physicians should consider and weigh the risk of hepatotoxicity vs. the risk of disease progression.
- Caution is required in patients initiated on a KRAS G12C inhibitor within 3 months post-ICI. These patients may require more frequent monitoring of liver function tests.

### 5.8. What Are the Potential Strategies to Overcome Treatment Resistance in Patients with KRAS G12C?

#### 5.8.1. Overview

Data from preclinical and clinical studies indicated that the efficacy of single-agent KRAS G12C-targeted therapy is hindered by drug resistance [108,109,110,111], which occurs relatively early, often a few months following treatment initiation [112]. This implies the need to better characterize the resistance pathways associated with KRAS G12C inhibitors [112,113].

To study the possible mechanisms of acquired resistance to adagrasib in the KRYSTAL-1 trial, histologic and genomic analyses (NGS on tissue or ctDNA) were performed, and pre-treatment samples were compared with those obtained after the development of resistance [114]. Samples from 38 patients were analyzed (27 NSCLC, 10 colorectal cancer, and 1 appendiceal cancer) and resistance to adagrasib was detected in 17 patients (45% of the cohort), of whom 7 (18% of the cohort) had multiple co-occurring mechanisms. The most frequent on-target mechanisms to adagrasib resistance included activating mutations in KRAS (G12D, G12V, G13D, Q61H), secondary KRAS mutations within the adagrasib-binding pocket and high-level amplification of the KRAS G12C allele. Acquired bypass mechanisms of resistance included activating mutations in other oncogenes, oncogenic fusions, loss-of-function mutations, and MET amplification. The data highlight the need for new therapeutic strategies to delay and overcome drug resistance. Nonetheless, an understanding of how one KRAS G12C inhibitor compares to another based on mechanisms of resistance with mutations in the switch II binding pocket might help determine the best sequence of therapies to overcome resistance.

In both CodeBreak100 and KRYSTAL-1 trials, KRAS inhibitor treatment led to favourable responses in patients with NSCLC harbouring STK11 mutation but lower responses in those with KEAP1 mutation [4,94]. Considering that STK11 mutation is associated with poor outcomes, adding a KRAS G12C inhibitor to first-line approaches may be an option. Furthermore, it has been suggested that KRAS inhibition can increase the susceptibility of tumour cells to ICI therapy by producing an inflammatory tumour microenvironment in tumours where specific co-mutations lead to a tolerogenic tumour-immune microenvironment [115].

Several ongoing trials are assessing combinations with KRAS G12C inhibitors and other therapies, including an ICI in the first-line setting. Preliminary data from the KRYSTAL-7 phase 2 trial and KRYSTAL-1 phase 1b cohort evaluating adagrasib (400mg twice daily) in combination with pembrolizumab as the first-line therapy for NSCLC harbouring a KRAS G12C mutation across all PD-L1 subgroups were presented at the 2022 ESMO Immuno-Oncology Annual Congress [94]. In 53 patients who were clinically evaluable and received at least one on-study scan, adagrasib–pembrolizumab demonstrated promising preliminary clinical activity across all PD-L1 subgroups with an ORR of 49%. Grade 3 increases in liver enzymes, reported in 9% of patients, confirmed that hepatotoxicity is indeed an emerging TRAE associated with KRAS G12C inhibitors. Similar to other AEs that emerged with novel therapeutic approaches, it is likely that with time and experience, clinicians will gain a better understanding and appreciation for KRAA G12C inhibitor-related hepatotoxicity and develop effective management strategies. Furthermore, as optimal management of KRAS-mutated NSCLC is expected to include combinations of targeted therapies and ICI, the safety profiles will play a significant role in how to move forward and sequence these therapies and which agents/combinations will advance in clinical development.

#### 5.8.2. Recommendations

- The panel strongly encourages the enrolment of patients with KRAS G12C mutated NSCLC into clinical trials with KRAS inhibitors.
- Currently repeated biopsy (liquid or tissue) in patients progressing on or after KRAS G12C inhibitors is not recommended outside clinical trial settings.

## 6. Conclusions

Similar to other driver mutations, timely detection and initiation of appropriate treatment are key to optimizing outcomes in patients with KRAS G12C. Thus, testing for KRAS G12C should be performed as part of a comprehensive panel that includes current standard-of-care biomarkers. Reflex biomarker testing for KRAS G12C could be initiated by the pathologist at the time of initial diagnosis, if NGS is performed.

KRAS G12C inhibitors are emerging as effective and safe options, although their sequencing among other available options and combinations with systemic therapies from other classes requires further study. Based on the current evidence, the panel recommends sotorasib, the only approved KRAS G12C inhibitor in Canada, over docetaxel for patients with locally advanced or metastatic KRAS G12C-mutated NSCLC upon progression on ICI(s) and/or chemotherapy. It is also reasonable to offer sotorasib as second-line therapy to patients who either progressed on ICI monotherapy that are not candidates for a platinum doublet or patients that received first-line chemotherapy that have a contraindication to ICIs. Although KRAS G12C inhibitors appear to have activity in brain metastases, there is insufficient evidence to recommend one agent over another or on how to sequence KRAS G12C inhibitors and radiation in patients with brain metastases.

KRAS G12C inhibitors can cause hepatotoxicity; therefore, regular monitoring of liver function is recommended. Clinicians should follow the drug’s product monograph for dose reductions, interruptions, or permanent discontinuations. Corticosteroids are recommended in patients presenting with grade 2–4 hepatotoxicity.

As there are still many unanswered questions implicated in therapeutic decision making (e.g., mechanisms of resistance, sequencing, use in combination with other therapies, management of toxicities), participation in clinical trials is recommended for patients with KRAS mutations. Upcoming results from ongoing clinical trials and the emergence of novel agents and combinations will further streamline the management of patients with KRAS-mutated NSCLC.

## Figures and Tables

**Table 1:** Efficacy of KRAS G12C Inhibitors in Phase 2 and 3 Clinical Trials.

Trial	Krystal-1 [94]	CodeBreak100 [4]	CodeBreak 200 [5]
Phase	2	2	3
Drug	Adagrasib	Sotorasib	Sotorasib
N	116	126	171
ORR	42.9% (95% CI 33.5–52.6)	37.1% (95% CI 28.6–46.2)	28.1% (95% CI 21.5–35.4)
DCR	79.5% (95% CI 70.8–86.5%)	80.6% (95% CI 72.6–87.2%)	82.5% (95% CI 75.9–87.8)
TTR	1.4 months (range 0.9–7.1 months)	1.4 months (range 1.2–10.1 months)	1.4 months (range 1.2–8.3 months)
DoR	8.5 months (95% CI 6.2–13.8)	11.1 months (95% CI 6.9-NE)	8.6 months (95% CI 7.1–18.0)
PFS, median	6.5 months (95% CI 4.7–8.4)	6.8 months (95% CI 5.1–8.2)	5.6 months (95% CI 4.3–7.8)
OS, median	12.6 months (95% CI 9.2–19.2)	12.5 months (95% 10-NE)	10.6 months (95% CI 8.9–14.0)
Follow up, median	12.9 months	15.3 months	17.7 months

ORR, overall response rate; DCR, disease control rate; TTR, time to response; DoR, duration of response; PFS, progression free survival; OS, overall survival.

**Table 2:** TRAEs with Sotorasib and Adagrasib Reported in >10% of Patients Treated With Both Agents in Phase 2 Clinical Trials.

Trial	Krystal-1 [94]		CodeBreak100 [4]	
Drug	**Adagrasib**		**Sotorasib**	
N	116		126	
Any TRAE	**Any Grade**	**Grade 3–4**	**Any Grade**	**Grade 3–4**
	113 (97%)	50 (43%)	88 (70%)	26 (21%)
Most Frequent TRAEs				
Diarrhea	73 (63%)	1 (<1%)	40 (32%)	5 (4%)
Nausea	72 (62%)	5 (4%)	24 (19%)	0
Fatigue	47 (41%)	5 (4%)	14 (11%)	0
Vomiting	55 (47%)	1 (<1%)	10 (8%)	0
ALT increased	32 (28%)	5 (4%)	19 (15%)	8 (6%)
AST increased	29 (25%)	4 (3%)	19 (15%)	7 (6%)
Decreased appetite	28 (24%)	4 (3%)	16 (13%)	0
Blood alkaline phosphatase increase	12 (10%)	4 (4%)	17 (14%)	1 (1%)
Peripheral edema	12 (10%)	0	18 (14%)	0
Anemia	21 (18%)	6 (5%)	18 (14%)	1 (1%)

TRAEs, treatment-related adverse events; ALT, alanine transaminase; AST, aspartate aminotransferase.

**Table 3:** Recommended Management of Sotorasib-related Hepatotoxicity.

Severity	Sotorasib Action	Medical Management, Monitoring and Follow-Up
Grade 2–4 AST or ALT	First occurrence: Withhold	Treat with corticosteroids ^a^ Closely monitor liver function tests Await resolution to baseline or grade 1 and resolution or improvement of hepatitis symptoms If applicable, restart at 1 dose level reduction
	Second occurrence: Withhold	Treat with corticosteroids ^a^ Closely monitor liver function tests Await resolution to baseline or grade 1 and resolution or improvement of hepatitis symptoms If applicable, restart at an additional 1 dose level reduction
	Third occurrence: Permanently discontinue	Treat with corticosteroids ^a^
AST or ALT > 3 ×ULN with totalbilirubin > 2 x ULN in the absence of alternative causes	Permanently discontinue	Treat with corticosteroids ^a^

AST, aspartate aminotransferase; ALT, alanine transaminase; ULN, upper limit of normal. Grading defined by National Cancer Institute Common Terminology Criteria for Adverse Events (NCI CTCAE) version 5.0. ^a^ Prednisone 0.25 to 1 mg/kg/day or equivalent, followed by taper. A low dose of prednisone may be considered for preventing another occurrence of hepatotoxicity upon re-challenge with a KRAS inhibitor. Recommended dose reduction levels for sotorasib: ● First dose reduction: 480 mg (4 tablets) once daily; ● Second dose reduction: 240 mg (2 tablets) once daily.

## Data Availability

The data discussed herein are available within the article and its references.
